# 

**Published:** 1920-11-13

**Authors:** 


### THE HOSPITAL

### The Modern Newspaper of Administrative Medicine and Institutional Life.

Telegraphic Address: OSPEDALE, RAND, LONDON. Telephone Number: 2734 GERRARD.

No. 1797, Vol. LXIX. | Saturday, November 13th, 1920. | PRICE TWOPENCE.

